# Synthesis of Benzimidazole-Sulfonyl Derivatives and Their Biological Activities

**DOI:** 10.1155/2022/7255299

**Published:** 2022-04-05

**Authors:** Endale Mulugeta, Yoseph Samuel

**Affiliations:** Department of Applied Chemistry, School of Applied Natural Science, Adama Science and Technology University, P.O.Box 1888, Adama, Ethiopia

## Abstract

Currently, the synthesis of new compounds with potential bioactivities has become a central issue in the drug discovery arena. Among these new compounds, benzimidazole-sulfonyl scaffolds have vital applications in the fields of pharmaceuticals industries. Benzimidazole and sulfonyl compounds have remarkable biological activities, such as antibacterial, antifungal, anti-inflammatory, antiproliferative, carbonic anhydrase inhibitory, and *α*-amylase inhibitory activities. Furthermore, recent literature mentions the synthesis and bioactivities of some benzimidazole-sulfonyl hybrids. In this review, we focus on reviewing the synthesis of these hybrid scaffolds and their various types of biological activities of the compounds.

## 1. Introduction

Disease resistance is one of the critical problems facing clinical repetition, and finding new effective compounds against multiresistant pathogens is one of the major goals in current biomedical research [1]. Among the heterocyclic compounds known in the literature for their various bioactivities are those with benzimidazole ring and those with sulfonamide moiety, which possess a wide range of medicinal properties. Usually, organic compounds with heterocyclic rings as the core of their chemical scaffolds have shown pharmaceutical activities. Furthermore, isolated natural products having heterocyclic rings are good candidates. Heterocyclic compounds provide distinct characteristics and promising applications in the pharmaceutical industry to discover, develop, and produce drugs. Nowadays, heterocyclic compounds are playing an important role to design and develop drugs with wide applications in the pharmaceutical area [2]. In this feature, benzimidazole anchored sulfonamide moiety has a substantial role in the bioactive compounds. Benzimidazole derivatives have developed a considerable interest in the medical field due to their therapeutic action as antimicrobial [3–6], antitumor [7–10], antihelmintic [11], antihistaminic [12–14], proton pump inhibitors [12, 15] anti-inflammatory [16–18], anticancer [19], antioxidant [20], and antihypertensive [21] drugs. Benzimidazole anchored with other heterocyclic compounds, such as triazole, thiadiazole, oxadiazole, morpholine, piperazine, or piperidine, has exhibited potential bioactivities [22, 23]. The presence of sulfonamide moiety within the heterocyclic template enriches the lipophilic properties of the developed derivatives [24].

**Figure 1:**
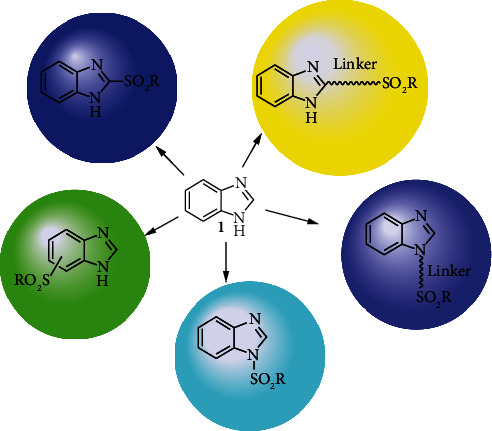
Chemical structure of benzimidazole-sulfonyl derivatives.

In this review, we focus on presenting various synthesis methods of benzimidazole-sulfonyl hybrid compounds and their biological activities such as antibacterial [25], antifungal [25], antiviral [25], antiproliferative [26], anti-inflammatory [27], carbonic anhydrase inhibitors [28], and alpha-amylase inhibitory [29]. The synthesis protocol of benzimidazole-sulfonamide compounds and their various bioactivities have not yet been well and adequately reviewed. Different scholars claim that the introduction of the sulfonamide moiety to the benzimidazole scaffold will enhance the antibacterial and antifungal activities of the synthesized compound [25]. The chemical structures of benzimidazole-sulfonyl derivatives are shown in Figure 1.

**Figure 2:**
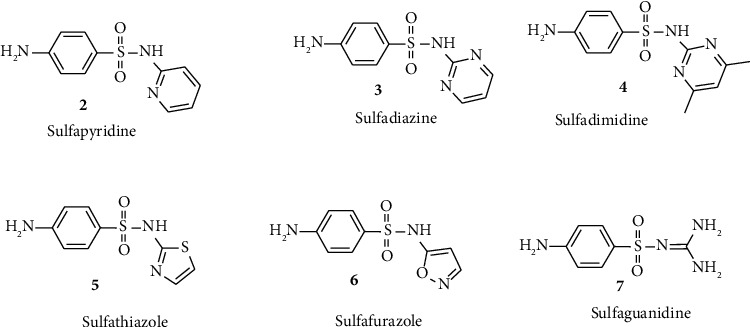
Chemical structures of sulfa drugs derivatives.

Sulfonyl-based drugs are recognized as sulfa drugs having sulfonyl moiety, and usually, they are employed for preventive and chemotherapeutic agents towards various bacterial infections [30]. Chemical structures of sulfa drugs with potential pharmaceutical activities having sulfonyl scaffolds such as sulfapyridine (2), sulfadiazine (3), sulfadimidine (4), sulfathiazole (5), sulfafurazole (6), and sulfaguanidine (7) are shown in Figure 2.

## 2. Synthesis of Benzimidazole-Sulfonyl Derivatives

**Figure 3:**
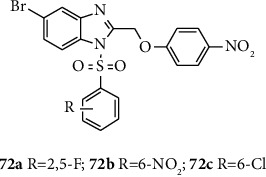
Chemical structures of antibacterial active benzimidazole-sulfonyl derivatives.

So far, various literature mention compounds having benzimidazole-sulfonyl scaffold have been prepared for their biological activities. Until now, researchers have tried and searched countless methodologies of the benzimidazole-sulfonyl drug-based compounds through improving the scaffold template. Sulfadiazine, sulfamoxole, and sulfathiazole antibacterial drugs with a sulfonyl moiety are the best-known candidates. Drugs having a sulfonyl scaffold usually show a broad range spectrum of bioactivities. Most compounds containing a sulfonyl moiety exhibited antibacterial activity [30], antifungal activity [31], antiviral activity [32], antiprotozoa activity [33], and anti-inflammatory activity [34] are well known. Moreover, they are used to prepare drugs for the remedy of Alzheimer's disease [35], rheumatoid arthritis [36], and obesity disease [37]. Recently, the synthesis of benzimidazole-sulfonyl hybrids scaffold and their biological activities has become more popular. Usually, the synthesis of benzimidazole has been conducted through the condensation reaction of *o*-phenylenediamine with carboxylic acids in the presence of an acid catalyst. Also, benzimidazole is prepared from condensation of *o*-phenylenediamine with a specific carbonyl compounds such as aldehyde, ketone, ester, amide, acid anhydride, and acid chlorides [38]. Frequently, to perform the condensation reaction, smooth acid catalysts such as hydrochloric acid, *p*-toluenesulfonic acid, or boric acid are needed commonly [38]. Benzimidazole-sulfonyl derivatives are prepared using the reaction of benzimidazole scaffold with sulfonamide derivatives at standard reaction conditions. Kumar and coworkers reported the synthesis and antimicrobial activities evaluation of novel benzimidazole-sulfonyl derivatives from moderate to excellent activities [39]. Here, compound 14 is prepared in an excellent yield with potential bioactive properties. In the beginning, the key structure 2-mercaptobenzothiazole (10) was prepared using *o*-phenylenediamine (8) and carbon disulfide (9) through double condensation reaction protocol at low pH (Figure 3).

**Scheme 1:**
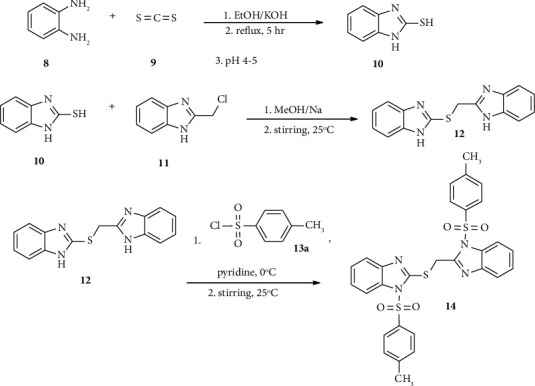
Synthetic scheme towards compound 14.

**Scheme 2:**
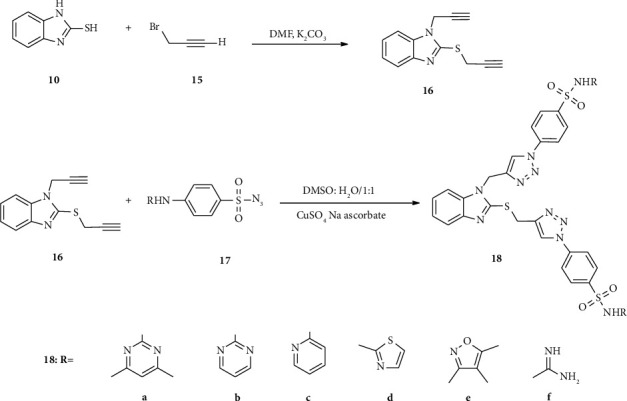
Synthetic routes towards 1, 2, 3-triazole benzimidazole-sulfonyl derivatives.

Later, compound 10 reacted with chloromethyl benzimidazole (11) moiety in methanol solvent to afford compound 12. Finally, compound 12 is subjected to *p*-toluene sulfonyl chloride (13a) in the pyridine solvent to provide the target compound 14 in a good yield. Al-blewi and coworkers' triazole-based benzimidazole-sulfonyl hybrids scaffolds synthesized (17a–f) are shown in Scheme 1. The synthesis reaction protocol contains two consecutive reaction steps. In the first step compound, 16 was synthesized using mercaptobenzothiazole (10) as starting material subjected to propargyl bromide (15) in the presence of K_2_CO_3_ in the DMF solvent. Then, an azide-alkyne cycloaddition click reaction type was carried out by mixing thiopropargylated benzimidazole (16) with the appropriate sulfa drug azide moiety 17 (a–f), in the presence of copper sulfate and sodium ascorbate additive to afford triazole-benzimidazole-sulfonyl hybrids (18a–f) in excellent yields [40] (Scheme 2 [41]).

**Scheme 3:**
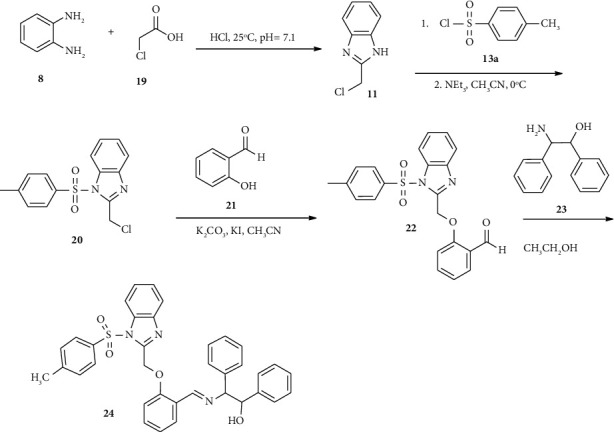
Synthetic routes towards compound 24.

New Schiff bases bearing benzimidazole and *p-*toluene sulfonyl moiety were synthesized and exhibited higher antimicrobial activities [42]. Commercially available *o*-phenylenediamine (8) condensed with *α*-chloroacetic acid (19) to afford compound 11 in a good yield. Subsequently, compound 11 was directly subjected to sulfonyl chloride 13a in the presence of triethylamine at 0°C to afford compound 20. Furthermore, compound 20 reacted with *α*-hydroxy benzaldehyde (21) in the presence of potassium carbonate and potassium iodide in acetonitrile solvent afford compound 22. Finally, compound 22 reacted with 2-amino-1, 2-diphenylethanol (23) in ethanol solvent to provide compound (Scheme 3).

**Scheme 4:**
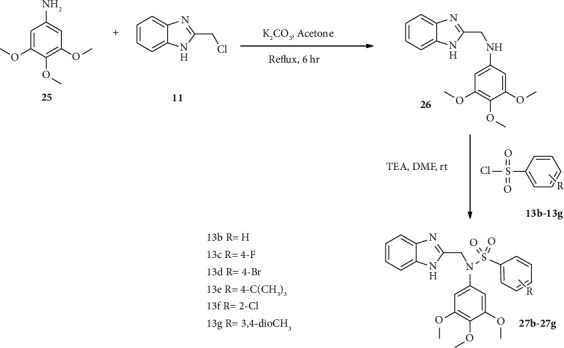
Synthetic routes towards compound 27.

Jian-Song and coworkers synthesized a series of benzimidazole-sulfonyl hybrids scaffold [27]. Here, aniline 25 reacted with benzimidazole 11 in the presence of potassium carbonate in acetone solvent to afford compound 26. Furthermore, compound 26 reacted with sulfonyl chloride 13 (b–g) in the presence of triethylamine in DMF solvent to provide benzimidazole-sulfonyl hybrid 27 as shown in Scheme 3 [27] (Scheme 4 [43]).

**Scheme 5:**
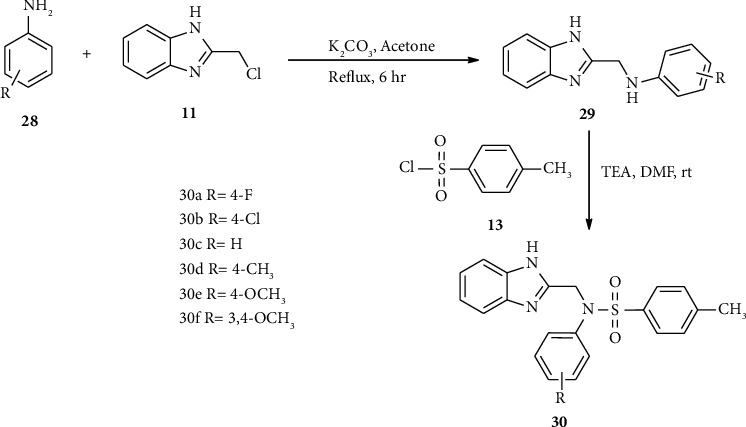
Synthetic routes towards compound 30.

According to the Jian-Song research group, the synthesis of compound 30 was accomplished via two consecutive substitution reaction steps as shown in Scheme 5.

With this reaction, compound 30 was synthesized via two consecutive reaction steps. Here, aniline 28 reacted with benzimidazole 11 in the presence of potassium carbonate in acetone solvent to afford compound 29. Compound 29 is directly subjected to sulfonyl chloride 13a in the presence of triethylamine in DMF solvent to afford the target compound 30 in a good to excellent yield [27].

**Scheme 6:**
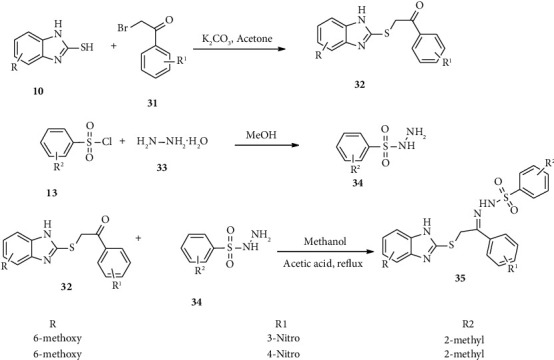
Synthetic routes towards compound 35.

Taha and coworkers synthesized benzimidazole-sulfonyl hybrid scaffold 35 with a potent *α*-amylase inhibitor [44]. Here, 2-mercaptobenzimidazole (10) refluxed with acyl bromide 31 in the presence of potassium carbonate in acetone to afford compound 32. Subsequently, sulfonyl chloride 13a was refluxed with hydrazine hydrate (33) in methanol solvent to afford compound 34. Finally, compound 32 reacted with tosyl hydrazine (34) in the presence of acetic acid and methanol affording compound 35 (Scheme 6) [44].

**Scheme 7:**
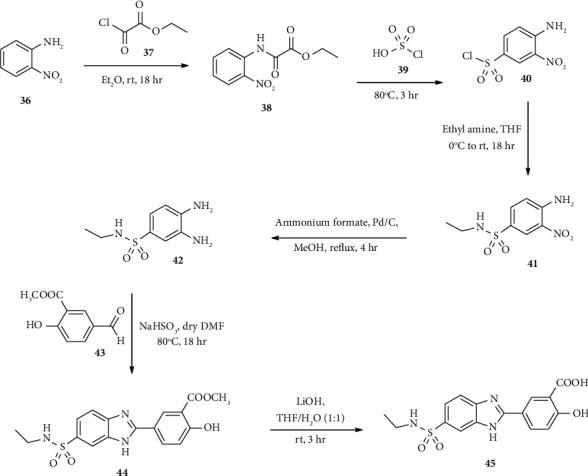
Synthetic routes towards compound 45.

Milite and coworkers synthesized benzimidazole-sulfonyl hybrid scaffold 45 as a core template with potent carbonic anhydrase inhibitors [45]. Here, aniline 36 was protected using acetate 37 in diethyl ether. The resulting intermediate compound 38 reacted with sulfonic acid 39 to afford sulfonyl chloride derivatives (40). Furthermore, compound 40 reacted with ethylamine in THF to afford nitrobenzene sulfonamide 41. Compound 41 was reduced through palladium/carbon catalyst in the presence of ammonium formate in methanol to afford the corresponding amino derivatives 42. Condensations of benzenesulfonyl 42 with aldehyde 43 in the presence of NaHSO_3_ afford compound 44. Finally, hydrolysis of compound 44 provides the target compound 45 (Scheme 7).

Tahlan and coworkers report the synthesis of a series of benzimidazole-sulfonyl derivatives 53 with potent antibacterial and antifungal activities [46]. This compound was prepared using four consecutive steps. In the first step, ethyl-2-(4-nitrophenoxy) acetate (48) was synthesized using the reaction of phenol (46) and ethyl-2-bromoacetate (47). Compound 48 treated with sodium hydroxide in ethanol was converted into the corresponding acid derivative 49.

**Scheme 8:**
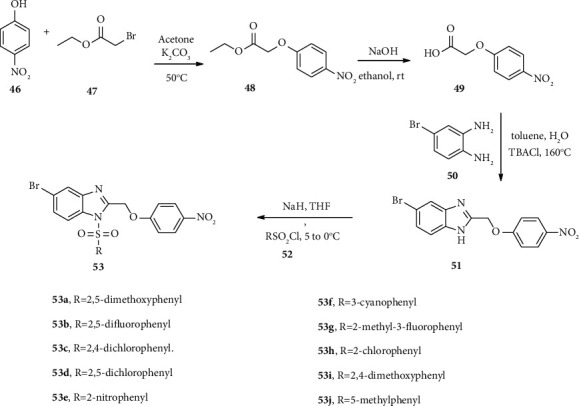
Synthetic routes towards compound 53 derivatives.

**Scheme 9:**
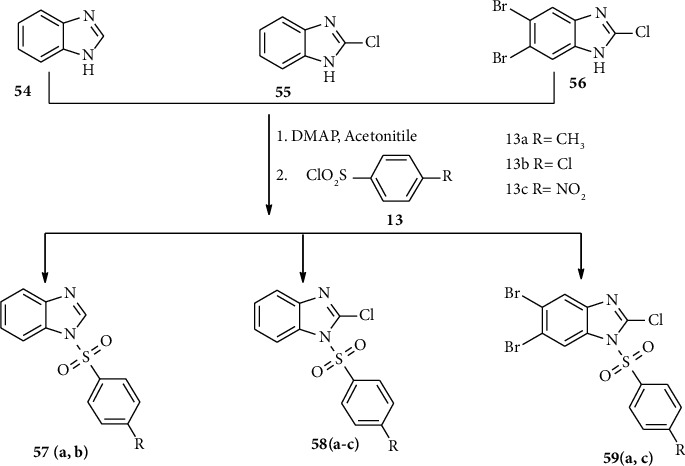
Synthetic routes towards compounds 57–59.

Compounds 49 and 50 through double condensation in the presence of tetrabutylammonium chloride salt provide benzimidazole derivative 51. Finally, through S_N_2 substitution reaction, compound 51 reacted with substituted sulfonyl chloride (52) affording benzimidazole derivatives 53a–j (Scheme 8). Naaz and coworkers synthesized new derivatives of benzimidazole-sulfonyl 57–59 with potential antibacterial activities [41]. Benzimidazole-sulfonyl derivative 57 was synthesized via S_N_2 substitution reaction of benzimidazole (54–56) and benzene sulfonyl chloride 13 (a–c) in the presence of DMAP as shown in Scheme 9.

**Scheme 10:**
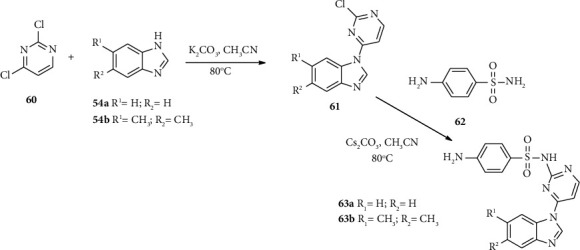
Synthetic routes towards compound 63 derivatives.

Sui and coworkers synthesized benzimidazole-sulfonyl hybrids with potent antibacterial and antifungal activities [47]. Compound 63 was synthesized from pyrimidine 60, benzimidazole 54, and *p*-amino benzenesulfonamide (62) according to the routes shown in Scheme 10.

**Scheme 11:**
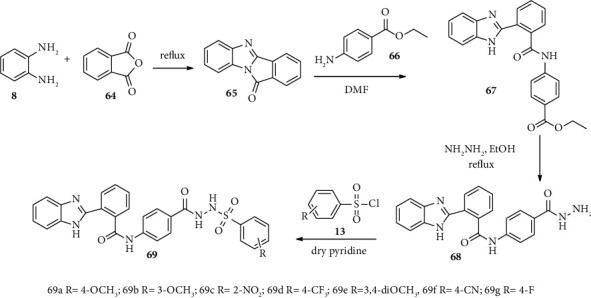
Synthetic routes toward compound 69 derivatives.

Mehta and coworkers synthesized novel benzimidazole derivatives with potential antibacterial and antifungal activities. Here, the target compound 69 was synthesized through three consecutive reaction steps. Compound 67 was synthesized from *o*-phenylenediamine 8 and anhydride 64 via cyclization reaction. Furthermore, compound 64 converted into compound 67 which is a benzimidazole moiety bearing acetamide substituent. Compound 67 undergoes a substitution reaction type with hydrazine to form benzimidazole bearing phenyl hydrazine 68. Finally, compound 68 and sulfonyl chloride 13 undergo substitution reaction to provide compound 69 in a good yield [43] (Scheme 11 [48]).

**Scheme 12:**
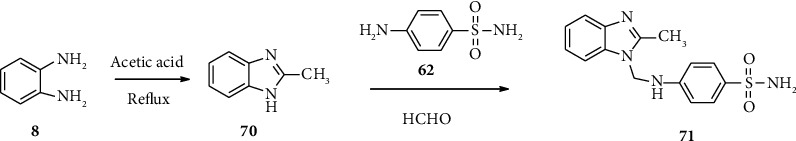
Synthetic routes towards compound **7**.

Rajasekha and coworkers synthesized biologically active benzimidazole-sulfonyl derivative 70 with potent anti-inflammatory activity [49]. Target compound 70 was synthesized via two steps reactions, in which condensation reaction of *o*-phenylenediamine (8) with acetic acid was followed by subjecting to compound 62 in the presence of formaldehyde through substitution reaction that affords compound 71 in good yields (Scheme 12) [50].

## 3. Bioactivities of Benzimidazole-Sulfonyl Scaffolds and Their Derivatives

Benzimidazole is heterocyclic aromatic compound with promising drug candidate and broad spectrum of biological activities. Benzimidazole anchored with sulfonyl moiety scaffold and derivatives have showed potent broad-spectrum activities. Here, antibacterial, antifungal, antiproliferative, antiviral, carbonic anhydrase activity, and alpha-amylase inhibitor activities possess benzimidazole-sulfonyl hybrids scaffold as templates for various structures that are briefly discussed [47].

### 3.1. Antibacterial Activities of Benzimidazole-Sulfonyl Derivatives

Benzimidazole-sulfonyl derivatives have exhibited potent antibacterial activity against various Gram-negative and Gram-positive bacterial strains. Compound 72 exhibited good antibacterial activities against Gram-positive and Gram-negative bacterial with MIC ranging from 0.1 to 0.50 mg/mL. The presence of the *p*-nitrophenyl methyl ether group at position 2 of N-toluene-sulfonyl bromobenzimidazole (72) and the antibacterial properties of the target compound exhibited good activity (Figure 3) [51].

### 3.2. Antifungal Activities of Benzimidazole-Sulfonyl Derivatives

**Figure 4:**
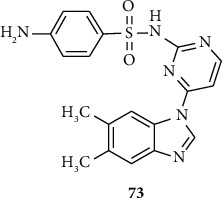
Chemical structures of antifungal active benzimidazole-sulfonyl derivatives.

Benzimidazole-sulfonyl hybrids also exhibited good antifungal activities [40]. Al-blewi and coworkers synthesized a series of sulfonyl amino benzimidazole derivatives 73 that exhibited good antifungal activities. The evaluation for their antimicrobial activities is against two fungal strains *Candida albicans* and *Aspergillus brasiliensis*, and it inhibited the fungal strains within MIC values range 32–64 *μ*g/mL (Figure 4) [40].

### 3.3. Antiviral Activities of Benzimidazole-Sulfonyl Derivatives

Tahlan and coworkers reported the synthesis and antiviral activities of novel benzimidazole 74a and 74b derivatives. Compounds 74a and 74b inhibited the growth of human immune deficiency virus type-1 (HIV-1) [46]. Other research groups, Chen and coworkers synthesized and report benzimidazole-sulfonamide derivatives of 75a–f to evaluate their antiviral activities.

**Figure 5:**
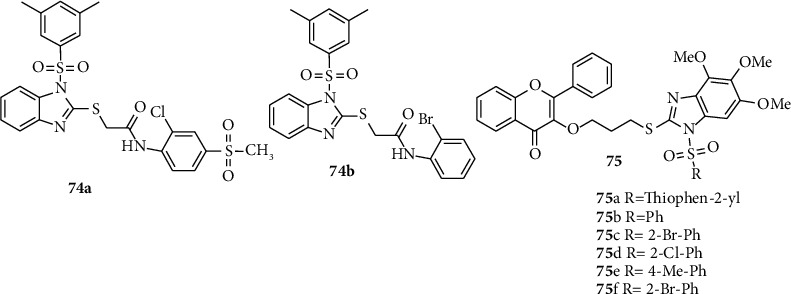
Chemical structures of antiviral active benzimidazole-sulfonyl derivatives.

Among these novel compounds, 75a and 75b showed antiviral activities impacts against TMV at 500 *μ*g/mL, with relating inhibitory paces of 61.5 and 57.6%, respectively, which were superior to that of ningnanmycin (55.8%) (Figure 5) [51].

### 3.4. Antiproliferative Activities of Benzimidazole-Sulfonyl Derivatives

**Figure 6:**
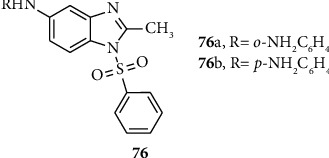
Chemical structures of antiproliferative active benzimidazole-sulfonyl derivatives.

Benzimidazole derivatives incorporated sulfonyl scaffolds exhibit antiproliferative activities against cancer cell. Shaharyar and Mazumder synthesized and report novel benzimidazole-sulfonyl derivatives 76 (a-b) with potent antiproliferative activities. Compounds 76 (a-b) were evaluated for their anti-inflammatory activities using acetic acid-induced writhing in mice where indomethacin was used as the standard drug and showed good antiproliferative activities (Figure 6) [52].

### 3.5. Anti-Inflammatory Activities of Benzimidazole-Sulfonyl Derivatives

**Figure 7:**
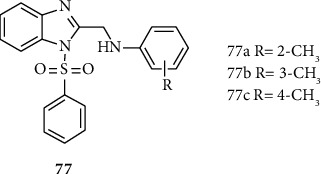
Chemical structures of anti-inflammatory active benzimidazole-sulfonyl derivatives.

Tahlan and coworkers synthesized and report anti-inflammatory active benzimidazole-sulfonyl derivatives 77 (a–c). Compounds 77 (a–c) showed good anti-inflammatory activities, compared to the standard drug indomethacin (Figure 7) [46].

**Figure 8:**
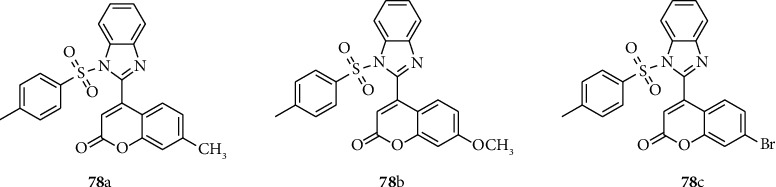
Chemical structures of anti-inflammatory active benzimidazole-sulfonyl derivatives.

Irfan and coworkers synthesized and report biologically active sulfonamide-coumarin-benzimidazole hybrids 78 (a–c). Among the synthesized compounds, sulfonyl-benzimidazole-coumarin platform 78a showed highly enhanced anticancer activity against HeLa human cervix disease cell line in correlation with the reference compound adriamycin (ADR) (Figure 8) [53].

### 3.6. Carbonic Anhydrases Inhibitors of Benzimidazole-Sulfonamide Derivatives

**Figure 9:**
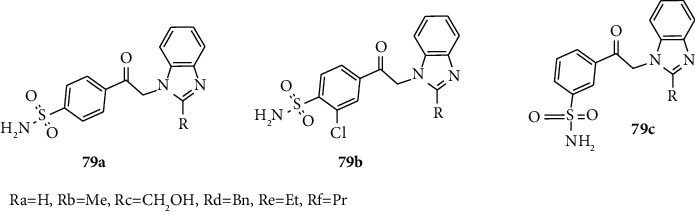
Chemical structures of carbonic anhydrases inhibitors active benzimidazole-sulfonyl derivatives.

Zubrien and coworkers synthesized and report sulfonyl anchored with benzene or benzimidazole rings 79 (a–f), and these derivatives act as inhibitors of carbonic anhydrases. Carbonic anhydrases are ubiquitous metalloenzymes, which catalyze the reversible hydration reaction of CO_2_ into protons and bicarbonate [53, 54]. Among these compounds, 79b showed the best inhibitors activities on CA I, II, VII, and XIII exhibiting affinities (Figure 9) [53, 54].

### 3.7. *α*-Amylase Inhibitory Activities of Benzimidazole-Sulfonyl Derivatives

**Figure 10:**
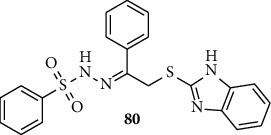
Chemical structure of *α*-amylase inhibitory active benzimidazole-sulfonamide derivative.

*α*-Amylase is hydrolyzed enzyme which comprises of Ca^+2^ ion in its active pocket and catalyzes the conversion of starch into monosaccharides glucose and maltose via hydrolysis using water [55]. Hussain and coworkers synthesized and report 2-mercaptobenzimidazole bearing sulfonyl scaffold. Compound 80 exhibited *α*-amylase inhibitor with IC_50_ value range 0.90 ± 0.05–11.2 ± 0.3 *μ*M, and the reference drug acarbose shows IC_50_ of 1.70 ± 0.10 *μ*M (Figure 10) [55].

## 4. Conclusions

In conclusion, this review summarizes the synthetic protocol of benzimidazole-sulfonyl hybrids and the potential bioactivity of the candidates such as antibacterial, antifungal, antiviral, antiproliferative, anti-inflammatory activity, carbonic anhydrase, and *α*-amylase inhibitory activities are discussed briefly. Thus, this review will serve as a good collection of literature in the field of pharmaceutical chemistry. A wide variety of benzimidazole derivatives have already been reported, and some are currently being used as active medicaments for the treatment of disease, for instance, wound healing. Still, various research groups are focusing on the development of newer benzimidazole-sulfonyl hybrids with better efficacy, potency, and lesser side effects. This area has got the tremendous potential to come up with new chemical entities of medicinal importance.
